# Hemoglobin Phenotype Distribution Among Future Healthcare Providers: A Descriptive Cross‐Sectional Study at a Ghanaian Health Sciences University

**DOI:** 10.1155/tswj/1199349

**Published:** 2026-01-20

**Authors:** Richard Vikpebah Duneeh, Debrah Sheila Yesuenam Ama, Mercy Adzo Klugah, Emmanuel Allotey, Elliot Elikplim Akorsu, Precious Kwablah Kwadzokpui, Kenneth Ablordey

**Affiliations:** ^1^ Department of Medical Laboratory Sciences, School of Allied Health Sciences, University of Health and Allied Sciences, Ho, Ghana, uhas.edu.gh; ^2^ Department of Medical Diagnostics, Kwame Nkrumah University of Science and Technology, Kumasi, Ghana, knust.edu.gh; ^3^ Department of General and Liberal Studies, School of Basic and Biomedical Sciences, University of Health and Allied Sciences, Ho, Ghana, uhas.edu.gh; ^4^ Department of Medical Laboratory, Ho Teaching Hospital, Ho, Ghana, hth.gov.gh

## Abstract

**Background:**

Abnormal hemoglobin phenotypes are prevalent genetic alterations in Ghana. Testing medical laboratory science students for these variants provides personal health information while enhancing their professional education as future healthcare providers. Thus, this study explored the hemoglobin phenotypes of medical laboratory science students at the University of Health and Allied Sciences (UHAS), Ho, Ghana.

**Methods:**

This study was a descriptive cross‐sectional study conducted among medical laboratory science students at the UHAS from July 2024 to August 2024. A data collection sheet was used to collate the sociodemographic characteristics such as ethnicity, town or place of origin, age, and gender of the participants. Venous blood samples of the study participants were drawn into ethylenediaminetetraacetic acid (EDTA) anticoagulated tubes. Hemoglobin variants of the samples were determined using the sickling test and alkaline hemoglobin electrophoresis method. Data was entered into a Microsoft Excel Spreadsheet and cleaned, then exported to IBM‐SPSS Version 27.0 (SPSS Inc., Chicago, Illinois, United States) for statistical analysis. A *p* value less than 0.05 was considered statistically significant.

**Results:**

Hemoglobin A was the most common phenotype, comprising 80.6% of the study population, followed by hemoglobin AS (10.9%) and hemoglobin AC (7.5%). No significant association was observed between hemoglobin phenotypes and participants′ regional origin, age, and sex.

**Conclusion:**

Hemoglobin A was the most prevalent phenotype among participants, with no significant links to age, sex, or region. The findings offer valuable baseline data and emphasize the need for future research exploring genetic, behavioral, and environmental factors shaping variant patterns.

## 1. Introduction

Hemoglobin, the oxygen‐carrying protein in red blood cells, is characterized by significant genetic diversity across human populations, a phenomenon reflecting evolutionary adaptations to environmental pressures like malaria [1]. In sub‐Saharan Africa, this diversity is exemplified by structural variants such as hemoglobin S (HbS), hemoglobin C (HbC), and hemoglobin E (HbE), which arise from single‐point mutations in the *β*‐globin gene and can alter the protein′s stability and function [2, 3]. The high prevalence of these variants in malaria‐endemic regions is a result of strong positive selection; for instance, the HbC allele reaches frequencies of nearly 20% in Northern Ghana and Burkina Faso, conferring a relative protection against *Plasmodium falciparum* malaria [3, 4]. In Ghana, newborn screening programs have identified a significant burden of these traits, with approximately 2% of babies born with sickle cell disease (SCD) and over 25% being carriers of an abnormal hemoglobin variant [5].

While the national prevalence is well‐documented in newborns and clinical cohorts [5], data on specific demographic subgroups, particularly educated youth, remain limited. This gap is significant as the distribution of hemoglobinopathies is influenced by regional endemicity and ethnic genetic backgrounds [3, 4]. Furthermore, carrier status has profound implications for reproductive health, yet awareness remains critically low. A study in Ghana′s Ahafo Region found that only 15.4% of young adults were knowledgeable about premarital carrier screening [6], a finding consistent with other reports that limited awareness contributes to high‐risk marriages and the birth of children with SCD [7, 8].

Future healthcare providers represent a strategically crucial demographic for addressing this public health challenge. They are not only at personal risk of being carriers but are also poised to become frontline professionals in diagnostics, transfusion safety, and patient education. A recent study among tertiary students in Cape Coast, Ghana, revealed that 15.5% carried a non‐A hemoglobin variant, underscoring the relevance of this population [9]. However, a focused analysis of medical laboratory science students, the very professionals who will conduct these tests and interpret results, is lacking.

Therefore, this study is aimed at determining the prevalence and sociodemographic distribution of hemoglobin variants among medical laboratory science students at the University of Health and Allied Sciences (UHAS). By focusing on this cohort, the study provides valuable baseline data to inform their professional training, enhance future genetic counseling and screening initiatives [6, 8], and support the development of targeted educational interventions within academic settings [10]. While mechanistic insights are beyond its scope, this work establishes a foundational prevalence dataset for future hypothesis‐driven research into the genetic and environmental factors shaping hemoglobinopathies in Ghana. By establishing this baseline data, our study is aimed at informing the integration of targeted education and screening programs within academic curricula, ultimately empowering this next generation of healthcare providers to lead future public health interventions [10].

## 2. Methodology

### 2.1. Study Design

This study was a descriptive cross‐sectional study which focused on determining hemoglobin variants among medical laboratory science students between July 2024 and August 2024.

### 2.2. Study Site

This study was conducted in the University Laboratory on the main campus of the UHAS, located in Ho, Ghana. The UHAS is the only public university in Ghana wholly dedicated to training healthcare professionals, offering programs in medicine, pharmacy, nursing, midwifery, and medical laboratory science, among others. Its student population is recruited nationally, providing unique students from diverse geographical and ethnic backgrounds across the country. The focus on medical laboratory science students, who are central to the diagnosis of hemoglobinopathies, makes them particularly relevant for the present study.

### 2.3. Study Population

The study population is made up of Level 100 to Level 400 undergraduate medical laboratory science students at the UHAS.

### 2.4. Inclusion Criteria

All medical laboratory science students at the UHAS who consented to participate in this study are included.

### 2.5. Exclusion Criteria

Medical laboratory science students were excluded if they were acutely ill, unavailable during the sampling period, or had a history of blood transfusion within the 3 months preceding the study.

### 2.6. Sample Size Determination

To determine the sample size, a study population of 418 was input into a Raosoft (2004) online calculator platform. Using a population size of 418, a minimum sample size of 201 was calculated; however, a sample size of 294 was used in this study to account for the nonresponse rate and to improve statistical power. A convenient sampling technique was employed in this study.

### 2.7. Sample and Data Collection

A data collection sheet was used to collect information on participants′ sociodemographic characteristics (age, sex, region, and level of program). Venous blood sample was aseptically taken from the participants and transferred into an ethylenediaminetetraacetic acid (EDTA) anticoagulant tube and labeled with a special identification number for each participant. Samples taken were kept on a rack and processed.

### 2.8. Determination of the Sickling Status and Hemoglobin Variants

The sodium metabisulfite test (sickling test) is a qualitative method used to induce polymerization and detect the presence of HbS under deoxygenated conditions. It is rapid, cost‐effective, and suitable for large‐scale screening, particularly in low‐resource settings. While highly sensitive for detecting HbS, it can rarely yield false‐negative results [11]. Complementing this, alkaline hemoglobin electrophoresis, performed at pH 8.6 on cellulose acetate, separates hemoglobin variants based on their electrophoretic mobility. This technique is a standard method for identifying common variants such as HbA, HbS, HbC, and HbF and is widely used in clinical and research settings due to its reliability [12].

### 2.9. Procedure for Sickling Test and Hemoglobin Alkaline Electrophoresis

**Figure 1:**
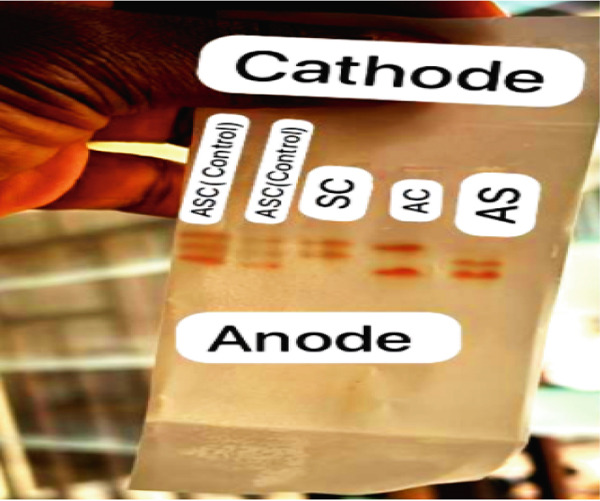
Alkaline hemoglobin electrophoresis (pH 8.6) on cellulose acetate. Hemoglobin electrophoresis was performed on cellulose acetate at alkaline pH to separate and identify different hemoglobin variants. The samples were applied close to the cathode and subjected to electrophoresis, allowing migration toward the anode based on charge differences. Known controls (ASC) were included alongside test samples (SC, AC, and AS) to ensure accuracy in band identification. Following electrophoresis, distinct hemoglobin bands were visualized and compared with controls for the interpretation of hemoglobin phenotypes.

The sickling test was performed by placing a drop of blood on a clean, grease free glass slide and an equal volume of 2% sodium metabisulfite solution added to induce deoxygenation. The mixture was gently mixed, cover‐slipped, and incubated at room temperature for 1 hour. After incubation, the slides were examined microscopically for sickled red blood cells; their presence indicated a sickling‐positive result, while absence indicated a negative result [13]. For hemoglobin variant analysis, alkaline hemoglobin electrophoresis was conducted using cellulose acetate paper. Hemolysate samples were applied to the moistened and tissue blotted acetate strip using an applicator and run in a Tris‐EDTA buffer system at pH 8.6. Electrophoresis was carried out at 250 V and 50 mA for 25 min, allowing separation of the hemoglobin variants based on their electrophoretic mobility [12]. A limitation of alkaline electrophoresis is its inability to reliably distinguish between variants with similar electrophoretic mobility, such as HbS and HbD or HbG. Confirmatory tests, such as acid electrophoresis or high‐performance liquid chromatography (HPLC), are required for definitive differentiation but were not employed in this study due to resource constraints (Figure 1).

### 2.10. Data Handling

Data collected from students was handled with the necessary care and confidentiality. Responses were not linked to their personalities, and all information was stored in a secure location. Only the investigators had access to the data acquired from the students.

### 2.11. Statistical Analysis

Data was entered into a Microsoft Office Excel 2021 spreadsheet. Descriptive and inferential analysis was done using IBM‐SPSS Version 26.0 (SPSS Inc., Chicago, Illinois, United States). Categorical outcomes were expressed as frequencies and proportions with 95% confidence intervals (CIs), which represent the estimated range of values within which the true population parameter is expected to lie. Hemoglobin phenotypes were dichotomized into normal (HbA and HbAF) and abnormal hemoglobin phenotypes (HbAS, HbAC, and HbC) to compute chi‐square, Fisher′s exact, and binary logistic regression (crude odds ratio) for age, sex, and region of origin. Age, sex, and region of origin were included to assess potential demographic and geographic differences in the distribution of hemoglobin phenotypes, and sex‐based patterns may reflect underlying population genetics. *p* values less than 0.05 were considered statistically significant.

### 2.12. Ethical Issues

The study received ethical approval from the UHAS Research and Ethical Committee (Ref: UHAS‐REC A10 [84] 23‐24), and permission was obtained from the University Laboratory managers. Informed consent was obtained from participants, who were assured of confidentiality and the right to withdraw at any time without penalty. Personal identifiers were removed, and data were coded and stored in a password‐protected file accessible only to the researchers. The data will be securely kept for 5 years for potential future research, with the same confidentiality measures applied.

## 3. Results

### 3.1. Sociodemographic Characteristics of Participants

A total of 294 participants were included in the study, with a median age of 21 years (range: 17–31). The majority (86.1%) were between 19 and 24 years old, with fewer participants aged ≤ 18 years (9.9%) and ≥ 25 years (4.1%). Males constituted 57.5% of the respondents, while females made up 42.5%. Most participants were in Level 200 (38.8%), followed by Level 100 (30.3%), Level 400 (21.1%), and Level 300 (9.9%). Regarding geographical distribution, 65.6% were from the southern zone, 25.5% from the middle zone, and 8.8% from the northern zone of Ghana (Table 1).

**Table 1:** Sociodemographic characteristics of participants.

**Variables**	**Frequency**	**Percentage (%)**
Total	294	100.0
Age		
Median (minimum–maximum)	21 (17–31)	
≤ 18	29	9.9
19–24	253	86.1
≥ 25	12	4.1
Sex		
Female	125	42.5
Male	169	57.5
Level of study		
100	89	30.3
200	114	38.8
300	29	9.9
400	62	21.1
Region of origin		
Northern zone	26	8.8
Middle zone	75	25.5
Southern zone	193	65.6

*Note:* Northern zone: Northern, Savannah, and Oti; middle zone: Bono, Ashanti, and Eastern; southern zone: Greater Accra, Central, Western, and Volta.

### 3.2. Sickling Status of Study Participants

**Figure 2:**
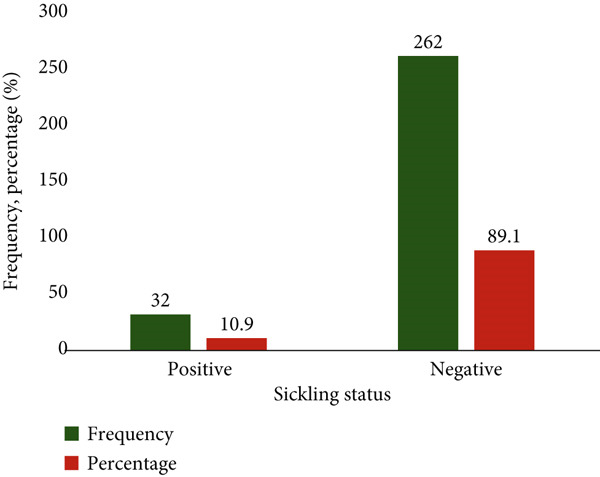
Sickling status among participants.

The figure illustrates the distribution of sickling status among the study participants. The study revealed that out of the 294 participants, 262 (89.1%) tested negative for sickling, while 32 (10.9%) tested positive (Figure 2).

### 3.3. Distribution of Hemoglobin Phenotype Among Study Participants

**Figure 3:**
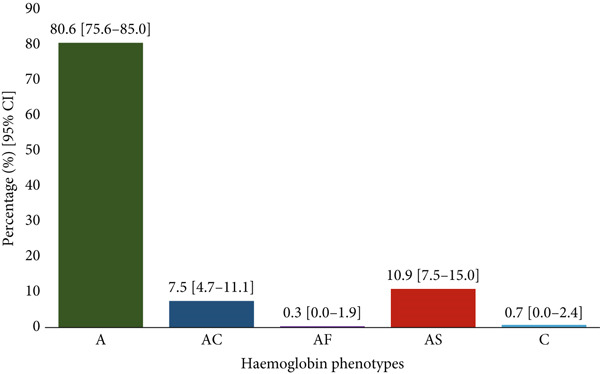
Distribution of hemoglobin phenotype among study participants.

The figure illustrates the distribution of hemoglobin phenotypes among study participants. All cases containing HbS were confirmed to be the heterozygous trait (HbAS); no homozygous SCD (HbSS) was identified. The most prevalent phenotype was hemoglobin A, accounting for 80.6% (95% CI: 75.6%–85.0%). Hemoglobin AS was the second most prevalent at 10.9% (95% CI: 7.5%–15.0%), followed by hemoglobin AC (HbAC) at 7.5% (95% CI: 4.7%–11.1%). The rarest phenotypes were AF at 0.3% (95% CI: 0.0%–1.9%) and C at 0.7% (95% CI: 0.0%–2.4%) (Figure 3).

### 3.4. Distribution of Hemoglobin Phenotype Stratified by Sociodemographic Characteristics

Table 2 presents the distribution of hemoglobin phenotypes (HbA, HbAC, HbAF, HbAS, and HbC) across different sociodemographic characteristics of the study participants. Overall, HbA was the most common phenotype (80.6%), followed by HbAS (10.9%) and HbAC (7.5%), with HbAF (0.3%) and HbC (0.7%) occurring rarely. Stratification by age revealed that HbA predominated across all groups, while HbAS was mainly observed among participants aged 19–24 years. With respect to sex, HbA was more frequent in both males and females, though HbAC and HbC were slightly more common in males. Distribution by level of study showed relatively similar patterns, with HbA being most prevalent, while HbAS was highest among fourth‐year students. Regional variation indicated that HbA remained predominant in all zones, though HbAS was slightly more frequent in participants from the southern and middle zones.

**Table 2:** Distribution of hemoglobin phenotypes across sociodemographic characteristics.

**Variables**	**HbA**	**HbAC**	**HbAF**	**HbAS**	**HbC**
	**n** **(%)**	**n** **(%)**	**n** **(%)**	**n** **(%)**	**n** **(%)**
Total	237 (80.6)	22 (7.5)	1 (0.3)	32 (10.9)	2 (0.7)
Age					
≤ 18	26 (89.7)	2 (6.9)	0 (0.0)	1 (3.4)	0 (0.0)
19–24	200 (79.1)	19 (7.5)	1 (0.4)	31 (12.3)	2 (0.8)
≥ 25	11 (91.7)	1 (8.3)	0 (0.0)	0 (0.0)	0 (0.0)
Sex					
Female	104 (83.2)	6 (4.8)	1 (0.8)	14 (11.2)	0 (0.0)
Male	133 (78.7)	16 (9.5)	0 (0.0)	18 (10.7)	2 (1.2)
Level of study					
100	72 (80.9)	8 (9.0)	1 (1.1)	8 (9.0)	0 (0.0)
200	92 (80.7)	8 (7.0)	0 (0.0)	12 (10.5)	2 (1.8)
300	25 (86.2)	2 (6.9)	0 (0.0)	2 (6.9)	0 (0.0)
400	48 (77.4)	4 (6.5)	0 (0.0)	10 (16.1)	0 (0.0)
Region of origin					
Northern zone	22 (84.6)	2 (7.7)	0 (0.0)	2 (7.7)	0 (0.0)
Middle zone	58 (77.3)	7 (9.3)	0 (0.0)	9 (12.0)	1 (1.3)
Southern zone	157 (81.3)	13 (6.7)	1 (0.5)	21 (10.9)	1 (0.5)

### 3.5. Sociodemographic Characteristics Associated With Hemoglobin Phenotypes

Overall, 19.0% of participants carried abnormal hemoglobin phenotypes. However, crude logistic regression analysis showed no statistically significant associations between hemoglobin phenotype status and age, sex, or region of origin (*p* > 0.05). Although abnormal phenotypes were relatively more common among males (21.3%) compared to females (16.0%) and among those aged 19–24 years (20.6%) compared to those aged ≤ 18 years (10.3%), these differences were not significant. Similarly, the prevalence of abnormal phenotypes did not vary meaningfully across the northern, middle, and southern zones (Table 3).

**Table 3:** Sociodemographic characteristics associated with hemoglobin phenotypes.

**Variables**	**Hemoglobin phenotypes**			
	**Normal Hb**	**Abnormal Hb**	**p** **value**	**cOR [95% CI]** **p** **value**
	**n** **(%)**	**n** **(%)**		
Total	236 (81.0)	56 (19.0)		
Age			0.261	
≤ 18	26 (89.7)	3 (10.3)		1
19–24	201 (79.4)	52 (20.6)		1.27 [0.12–13.53] 0.844
≥ 25	11 (91.7)	1 (8.3)		2.85 [0.36–22.55] 0.322
Sex			0.160	
Female	105 (84.0)	20 (16.0)		1
Male	133 (78.7)	36 (21.3)		0.70 [0.39–1.29] 0.254
Region of origin			0.616	
Northern zone	22 (84.6)	4 (15.4)		1
Middle zone	58 (77.3)	17 (22.7)		0.82 [0.27–2.53] 0.731
Southern zone	158 (81.9)	35 (18.1)		1.32 [0.69–2.54] 0.401

*Note:* *p* < 0.050 was considered statistically significant.

Abbreviation: cOR, crude odds ratio.

## 4. Discussion

Our study provides a contemporary perspective on hemoglobin variant distribution among medical laboratory science students at the UHAS in Ghana. The prevalence of HbA in our study aligns with findings from other studies in Cape Coast, Ghana, and Akwa Ibom state in Nigeria [9, 14]. A population‐based newborn screening study in Ghana has consistently shown that HbA remains the most prevalent genotype, with HbAS and HbAC present in smaller proportions [5]. This reflects both the natural distribution of hemoglobin variants in the population and the survival advantage of individuals with HbA in regions where hemoglobinopathies can be detrimental if not detected early.

Our results showed that 10.9% of participants carried the sickle cell trait (HbAS), while 7.5% carried HbAC. These findings are consistent with a population‐based study from Northern Ghana, which also identified HbAS as the second most common hemoglobin variant, followed by HbAC, a pattern attributed to malaria endemicity and interethnic admixture in the region [4]. At the national level, the prevalence of HbAS has been estimated at approximately 25%–30%, indicating that our observed prevalence is slightly lower but still within the expected range for Ghana [15]. Comparable results have been documented in other Ghanaian studies: Antwi‐Baffour et al. [15] reported 11.3% HbAS, Abdul et al. [16] observed 13.3% HbAS and 12.7% HbAC, and Ansah et al. [17] reported 12.9% HbAS and 7.7% HbAC among blood donors.

This consistency is biologically and epidemiologically sound, as the selective pressure exerted by endemic *Plasmodium falciparum* malaria, which historically influenced the persistence of these alleles, is not restricted by educational attainment or socioeconomic background [4]. Therefore, even among tertiary‐educated individuals from diverse regions and ethnicities, these hemoglobin variants remain prevalent due to shared ancestral genetic traits and evolutionary pressures. Although carriers of HbAS and HbAC are typically asymptomatic, they still risk passing abnormal hemoglobin alleles to their children, resulting in conditions such as HbSS, HbCC, or HbSC. Alarmingly, a recent study in Ghana′s Ahafo Region found that only 15.4% of young adults were knowledgeable about premarital carrier screening [6]. This stark awareness gap represents a missed opportunity for prevention. Other studies have similarly shown that limited awareness of carrier status contributes to high‐risk marriages and increases the likelihood of children being born with SCD [7, 8]. By targeting health science students, future frontline professionals in diagnostics, transfusion, and health education, we have a strategic chance to boost community‐wide awareness.

Notably, the HbAC prevalence among male students (9.5%) was nearly double that of female students (4.8%), although the difference was not statistically significant. While this sex‐based disparity could result from sampling variability, it also opens questions about possible sex‐linked biological differences in globin gene expression, health‐seeking behavior, or sociocultural mating patterns. Although HbE has been reported in other populations [3], it was not detected in our study, suggesting that this variant is either rare or absent in the studied population. A recent study has shown that sex influences hemoglobinopathy awareness, screening uptake, and even clinical outcomes [10]. Further investigation is needed to determine whether these sex‐linked trends are biologically or socially driven.

The study also highlights several areas for future hypothesis‐driven research. For instance, does being in a health science training program improve awareness of hemoglobin status? Could the hemoglobin variant patterns in this group be influenced by regional selection pressures, such as historical malaria exposure or tribal inheritance patterns? Previous studies have demonstrated regional clustering of HbC in Northern Ghana and Burkina Faso, partly due to the protective effects against *Plasmodium falciparum* and ethnogenetic variation [3, 4].

With regard to confounding variables, no significant associations were found between hemoglobin variant type and participants′ age, sex, or regional origin. However, this does not exclude the potential influence of unmeasured factors such as ethnicity, parental consanguinity, or local malaria prevalence.

## 5. Conclusion

This study fills a critical knowledge gap by documenting hemoglobin variant distribution among medical laboratory trainees at the UHAS. Although not mechanistically oriented, it highlights key allelic patterns that align with regional epidemiology and flags compelling avenues for future research into gene–environment interactions, education, and policy. We believe these findings can catalyze targeted public health interventions and genetic counseling strategies within academic and clinical settings in Ghana.

## 6. Limitations of This Study

This study has several limitations. Firstly, it was observational and cross‐sectional, limiting causal inferences and mechanistic insights. Secondly, while the sodium metabisulfite test is a valuable screening tool, it is known to be susceptible to false‐negative results due to factors such as high levels of HbF or improper deoxygenation. However, it is noteworthy that all electrophoretically confirmed HbAS cases in this study were also positive by the sickling test. Thirdly, the quantification of variant proportions was not performed due to a lack of access to densitometric analysis equipment. While alkaline electrophoresis reliably enabled phenotypic identification, this limitation precludes further quantitative analysis of the variants. Also, the sample may not fully represent Ghana′s genetic diversity, affecting generalizability. Finally, clinical outcomes were not assessed, and the low number of individuals with HbS and HbC variants may have reduced statistical power to detect associations.

## 7. Recommendations

Based on the study′s findings and limitations, future research should prioritize a multiphase approach. A university‐wide investigation involving students from multiple colleges (e.g., nursing, medicine, pharmacy, and public health) at the UHAS is essential. Employing a stratified random sampling design would yield representative data on hemoglobin variant distribution and awareness, directly expanding upon the foundational insights of this focused departmental study. Subsequently, longitudinal designs could elucidate causal relationships and temporal trends, while incorporating molecular analyses would uncover underlying genetic mechanisms. Expanding sampling to broader geographic and ethnic populations would enhance generalizability, and linking specific genotypes to clinical outcomes could better inform patient management strategies. Ultimately, these research efforts should parallel and support strengthened public education and early screening programs to improve the detection and management of hemoglobinopathies across Ghana.

## Conflicts of Interest

The authors declare no conflicts of interest.

## Author Contributions

**Conceptualization**: Richard Vikpebah Duneeh; **methodology**: Elliot Elikplim Akorsu; **formal analysis**: Kenneth Ablordey and Precious Kwablah Kwadzokpui; **resources**: Debrah Sheila Yesuenam Ama and Emmanuel Allotey; **project administration**: Debrah Sheila Yesuenam Ama; **investigation**: Debrah Sheila Yesuenam Ama; **writing—original draft**: Kenneth Ablordey and Precious Kwablah Kwadzokpui; **writing—review and editing supervision**: Richard Vikpebah Duneeh, Emmanuel Allotey, Mercy Adzo Klugah, and Elliot Elikplim Akorsu.

## Funding

No funding was received for this manuscript.

## Data Availability

The data used for this study will be provided upon request.
